# Anti-Inflammatory Mechanisms of Koreanaside A, a Lignan Isolated from the Flower of *Forsythia koreana*, against LPS-Induced Macrophage Activation and DSS-Induced Colitis Mice: The Crucial Role of AP-1, NF-κB, and JAK/STAT Signaling

**DOI:** 10.3390/cells8101163

**Published:** 2019-09-27

**Authors:** Tae-Woo Kim, Ji-Sun Shin, Kyung-Sook Chung, Yeong-Geun Lee, Nam-In Baek, Kyung-Tae Lee

**Affiliations:** 1 Department of Pharmaceutical Biochemistry, College of Pharmacy, Kyung Hee University, Seoul 02447, Korea; marle1@hanmail.net (T.-W.K.); jsshin@khu.ac.kr (J.-S.S.); adella76@hanmail.net (K.-S.C.); 2 Department of Life and Nanopharmaceutical Sciences, Graduate School, Kyung Hee University, Seoul 02447, Korea; 3 Natural Product Chemistry Laboratory, Graduate School of Biotechnology, Kyung Hee University, Yongin 17104, Korea; lyg629@nate.com (Y.-G.L.); nibaek@khu.ac.kr (N.-I.B.)

**Keywords:** KA, AP-1, NF-κB, JAK/STAT, RAW 264.7 macrophages, DSS-induced colitis

## Abstract

The current treatment options for inflammatory bowel disease (IBD) are unsatisfactory. Therefore, novel and safer therapies are needed. We previously reported that koreanaside A (KA) showed high radical scavenging activity and suppressed vascular cell adhesion molecule 1 (VCAM-1) expression in vascular smooth muscle cells. However, the molecular mechanisms involved in its anti-inflammatory effect have not been reported. KA inhibited pro-inflammatory mediators such as inducible nitric oxide synthase (iNOS), cyclooxygenase-2 (COX-2), nitric oxide (NO), and prostaglandin E_2_ (PGE_2_). KA inhibited the production and mRNA expression of interleukin (IL)-6 and tumor necrosis factor-α (TNF-α) induced by LPS. KA downregulated the myeloid differentiation primary response 88 (MyD88)-dependent inflammatory gene expressions in the MyD88-overexpressed cells. KA suppressed the LPS-induced transcriptional and DNA-binding activities of activator protein-1 (AP-1) and nuclear factor-kappa B (NF-κB). KA was found to inhibit the phosphorylation of Janus kinase 1/2 (JAK1/2) and signal transducers and activators of transcription 1/3 (STAT1/3). In DSS-induced colitis mice, KA relieved the symptoms of colitis by suppressing inflammatory cell infiltration, restoring tight junction (TJ)- and epithelial–mesenchymal transition (EMT)-related protein expression, and inactivating AP-1, NF-κB, and STAT1/3. Therefore, KA reduced inflammatory responses by downregulating AP-1, NF-κB, and JAK/STAT signaling in LPS-induced macrophages and DSS-induced colitis mice.

## 1. Introduction

Inflammatory bowel disease (IBD) commonly manifests in the form of Crohn’s disease and ulcerative colitis. The pathogenesis of IBD may be characterized by a loss of intestinal epithelial integrity and a dysregulated immune system [1,2]. The intestinal tight junction (TJ) is composed of several proteins including transmembrane proteins such as zona occludens (ZO)-1, occludin, claudins, and junctional adhesion molecule (JAM). These proteins may play a key role in epithelial barrier regulation [3]. In addition, the epithelial–mesenchymal transition (EMT) is a process characterized by the acquisition of a fibroblast-like elongated morphology, loss of cell polarity, enhanced migration ability, greater invasive capacity, and loss of epithelial markers [4]. Several studies have reported that the disruption of the physiological conditions of the TJ and EMT network can contribute to the pathogenesis of ulcerative colitis and alter intestinal permeability in IBD. Therefore, the restoration of TJ and EMT protein expression is a possible therapeutic strategy to improve the clinical outcomes of IBD [5,6]. Additionally, pro-inflammatory cytokines including tumor necrosis factor-α (TNF-α), interferon gamma (IFN-γ), interleukin (IL)-6, IL-12, IL-17, and anti-inflammatory cytokines such as IL-4, IL-10, and transforming growth factor-beta (TGF-β) play an important role in the regulation of inflammation [7,8].

Macrophages are the main sources of pro-inflammatory mediators in acute and chronic inflammatory diseases [9]. The activation of toll-like receptors (TLRs) on the surface of macrophages triggers downstream signaling involving the myeloid differentiation primary response 88 (MyD88) protein and TGF-β-activated kinase 1 (TAK1)/TAK1-binding (TAB) protein complex, leading to the activation of the activator protein-1 (AP-1) and the nuclear factor-kappa B (NF-κB) pathway [10,11]. The regulation of AP-1 is associated with the activation of mitogen-activated protein kinases (MAPKs), which play an essential role in signal transduction by modulating gene transcription. They include extracellular signal-regulated protein kinase (ERK), c-Jun N-terminal kinases (JNK), and p38. Activated MAPKs modulate the activity of the synthesized proteins c-Jun and c-Fos, which can dimerize to form the AP-1 complex and regulate inflammation by promoting the expression of pro-inflammatory mediators [12,13]. The activation of NF-κB is regulated by TAK1-mediated IκB kinase (IKK) complex activation. IKK phosphorylates the inhibitor of κB (IκBα) at two N-terminal serine residues (Ser32 and Ser36) and triggers ubiquitin-dependent IκBα degradation in the proteasome [14]. As a result, NF-κB translocates to the nucleus, where it binds to promoter regions of the target gene and induces pro-inflammatory mediators such as iNOS, COX-2, TNF-α, and IL-6 [15]. The Janus kinase/signal transducers and activators of the transcription (JAK/STAT) signaling pathway are involved in the pathogenesis of autoimmune and inflammatory diseases. Activated JAK proteins result in the transphosphorylation or autophosphorylation of the associated JAKs, and lead to the JAK-mediated tyrosine phosphorylation of STAT proteins [16]. Once activated, STAT proteins are dissociated from the receptor, homodimerized or heterodimerized, and translocated from the cytosol into the nucleus to modify the specific regulatory sequence of target genes such as iNOS and COX-2 [17].

*Forsythia koreana*, a flowering plant in the family Oleaceae, is distributed in East Asia. The fruits of *F. koreana* (*Fructus Forsythiae*; Korean name, ‘Yeon-kyo’) have been reported to exhibit various pharmacological effects such as anti-oxidant, anti-allergic, and anti-bacterial effects [18,19,20]. *F. koreana* contains metabolites such as lignans, flavonoids, phenylethanoids, and sterols [21]. Although studies of *Fructus Forsythiae* have been actively conducted, studies with a focus on the pharmacological activity of ingredients from the flowers of *F. koreana* are limited. Koreanaside A (KA), a lignan isolated from the flowers of *F. koreana*, has been reported to exert anti-oxidant activity and inhibitory effects on vascular cell adhesion molecule 1 (VCAM-1) expression in TNF-α-stimulated mouse vascular smooth muscle (MOVAS) cells [22]. However, the molecular mechanisms involved in its anti-inflammatory effect have not been reported. Therefore, we investigated the anti-inflammatory potential and molecular mechanisms of KA in LPS-stimulated RAW 264.7 macrophages and dextran sulfate sodium (DSS)-induced mice.

## 2. Materials and Methods

### 2.1. Plants Material and Isolation of Koreanasdie A (KA)

The flowers of *F. koreana* were gathered from Kyung Hee University global campus (Yong-In, Republic of Korea) in April 2015 and identified by Dae-Keun Kim, College of Pharmacy, Woosuk University (Jeonju, Republic of Korea). A voucher specimen (KHU-NPCL-201504) has been deposited with the Laboratory of Natural Products Chemistry, Kyung Hee University. The KA used for the present study was isolated as previously described [22]. KA was purified using Prep-HPLC equipment (a Waters 600S with Waters 2487 UV detector, 280 nm, Milford, MA, USA). The column was a Kinetex C18 column (Phenomenex, 5 μm, 250 × 4.6 mm). The mobile phase (0.1% FA in water, solvent A; acetonitrile, solvent B) was eluted at a flow rate of 0.4 mL/min with the following elution gradient of B: 5% (0.01 min) → 13% (5 min) → 13% (15 min) → 17% (18 min) → 17% (20 min) → 25% (14 min) → 100% (37 min) → 100% (40 min). HPLC-grade acetonitrile and water were purchased from Burdick & Jackson (Muskegon, MI, USA). Data acquisition and processing were done with Empower Waters software. As a result, KA, purity above 97%, was obtained by further purification using Prep-LC system equipped with ODS column.

### 2.2. Cell Culture and Treatment

RAW 264.7 macrophages were obtained from the Korean Cell Line Bank (Seoul, Republic of Korea). Mouse peritoneal macrophage cells were obtained 4 days after the intraperitoneal injection of 2 mL of thioglycollate to the 10-week-old C57BL/6 male mice and isolated as reported previously [2]. Cells were treated with KA (20, 40, or 80 μM) or with suitable positive control, and then stimulated with lipopolysaccharide (LPS) (1 μg/mL) for the incubated time.

### 2.3. Cell Viability Assay

After incubation with KA for 24 h, cells were treated with an 3-(4,5-Dimethylthiazol-2-yl)-2,5-Diphenyltetrazolium Bromide (MTT) solution for 4 h at 37 °C. MTT formazan were dissolved by adding dimethyl sulfoxide, and the absorbance of each well at 540 nm was read by a microplate reader (Molecular Devices, Sunnyvale, CA, USA).

### 2.4. Determination of NO, PGE_2_, TNF-α, and IL-6 Production

RAW 264.7 cells were incubated with KA (20, 40, or 80 μM) 1 h prior to LPS (1 μg/mL) stimulation for 6 h (IL-6 and TNF-α) and 24 h (NO and PGE_2_). The supernatant was collected, and nitrite levels in culture media were detected using Griess reaction and presumed to reflect NO levels. Culture medium (100 µL) was mixed with 100 µL of Griess reagent [equal volumes of 1% (*w*/*v*) sulfanilamide in 5% (*v*/*v*) phosphoric acid and 0.1% (*w*/*v*) (naphtylethylenediamine-HCl) for 10 min. Next, the absorbance of mixture was measured at 540 nm by a micromultiplate reader. The amount of nitrite in the samples was determined with reference to a sodium nitrite standard curve. PGE_2_, TNF-α, and IL-6 levels in cell culture media were quantified by PGE_2_ (Enzo Life Sciences, Inc., Farmingdale, NY, USA) and TNF-α and IL-6 (BD Bio-science, Sand Diego, CA, USA) enzyme immunoassay (EIA) kits according to the manufacturer’s instructions.

### 2.5. Western Blot Analysis

Western blot analysis was determined as described previously [23]. Briefly, cells or segment of the colon were homogenized or resuspended in PRO-PREP protein extraction solution (Intron biotechnology, Seoul, Korea) and then incubated for 30 min at 4 °C. Blots were again washed three times with Tween 20/Tris-buffered saline and then developed by enhanced chemiluminescence solution.

### 2.6. Plasmid, Transient Transfection, and Luciferase Assay

The transfection with pGL3-iNOS, pGL3-COX-2, pAP-1-Luc, and pNF-κB-Luc and luciferase assay were carried out as described previously [23]. V5-MyD88 vector was a kind gift from Kyung-Soo Inn (Kyung Hee University, Republic of Korea). RAW 264.7 macrophages were transfected with expression vectors encoding V5-MyD88 using Lonza Nucleofector (Basel, Switzerland). After 48 h of transfection, cells were treated with KA (20, 40, or 80 μM) for 2 h and then subjected to the Western blot analysis or qRT-PCR.

### 2.7. RNA Preparation and qRT-PCR

qRT-PCR was determined as described previously [24]. The oligonucleotide primers are described in Supplementary Table S1.

### 2.8. Nuclear Extraction and Electrophoretic Mobility Shift Assay (EMSA)

Cells were pretreated with KA for 1 h, and then stimulated with LPS (1 μg/mL) for indicated times. Nuclear extraction and the analysis of binding activity to biotin-labeled NF-κB or AP-1 oligonucleotides were conducted as described previously [23].

### 2.9. Experimental Animals

All animal experiments were conducted under university guidelines and were approved by the ethical committee for Animal Care and Use of Kyung Hee University in accordance with the animal protocol (Approval number # KHPASP(SE)-18-160), as described previously [24]. Male ICR mice weighing 20–25 g were purchased from the Orient Bio Inc. (Seongnam-si, Korea).

### 2.10. Induction of Colitis

Experimental colitis was induced by giving mice purified drinking water containing 4% (*w*/*v*) DSS (MW 36,000–50,000 Da, MP biomedicals, Inc Irvine, CA, USA) for 7 days ad libitum. The mice in each group were monitored carefully every day to confirm that they consumed an approximately equal volume of DSS-containing water. For each experiment, the mice were divided into five experimental groups (*n* = 12), and they were given different treatments: (1) mice drinking normal water and receiving vehicle orally per oral (p.o.) once daily (vehicle-treated control group); (2) mice drinking DSS water and receiving vehicle orally (p.o.) once daily (DSS-treated group); (3) mice drinking DSS water and receiving 5-ASA (75 mg/kg/day p.o.) daily (DSS + 5-ASA-treated group); (4) and (5) mice drinking DSS water and receiving KA (5 or 20 mg/kg, intraperitoneal (i.p.)) daily (DSS + KA 5 or 20 mg/kg-treated group). In our preliminary toxicity studies in normal mice, no toxicities were observed with intraperitoneal injection of KA once a day for 7 days up to 20 mg/kg/day. All materials were dissolved in sterilized 0.9% saline, and the administration of each drug was started at the same time as the DSS treatment. Body weight, stool consistency, and gross bleeding were recorded daily. The disease activity index (DAI) was determined by averaging the scores of body weight loss, stool consistency, and gross bleeding. Each score was determined as follows: change in body weight loss (0: none, 1: 1–5%, 2: 5–10%, 3: 10–20%, 4: >20%), stool blood (0 = no blood, 2 = blood trace in the stool clearly visible, 4 = gross rectal bleeding), and stool consistency (0: normal, 1 and 2: loose stool, 3 and 4: diarrhea). The mice were sacrificed at the end of the experiment, and the colons were separated. The colon length was measured between the ileo–cecal junction and the proximal rectum. The spleens were also obtained, and their weight was measured.

### 2.11. Histopathology

The resected large intestine was grossly examined for mucosal defects, hemorrhage, or ulcerative lesions, and it was immediately fixed in 4% paraformaldehyde overnight and embedded in paraffin. Histopathology and immunohistochemical staining were performed by Korea Experimental Pathology Inc. (Gyeonggi-do, Korea). Histopathological analysis was determined as described previously [24].

### 2.12. Statistical Analysis

The results are expressed as the mean ± SD of in vivo (*n* = 12) and in vitro triplicate experiments. Statistically significant values were compared using ANOVA and Dunnett’s post hoc test, and *P*-values of less than 0.05 were considered to be statistically significant.

## 3. Results

### 3.1. KA Inhibits NO and PGE_2_ Production through the Suppression of iNOS and COX-2 Expression in LPS-Induced RAW 264.7 and Peritoneal Macrophages

**Figure 1:**
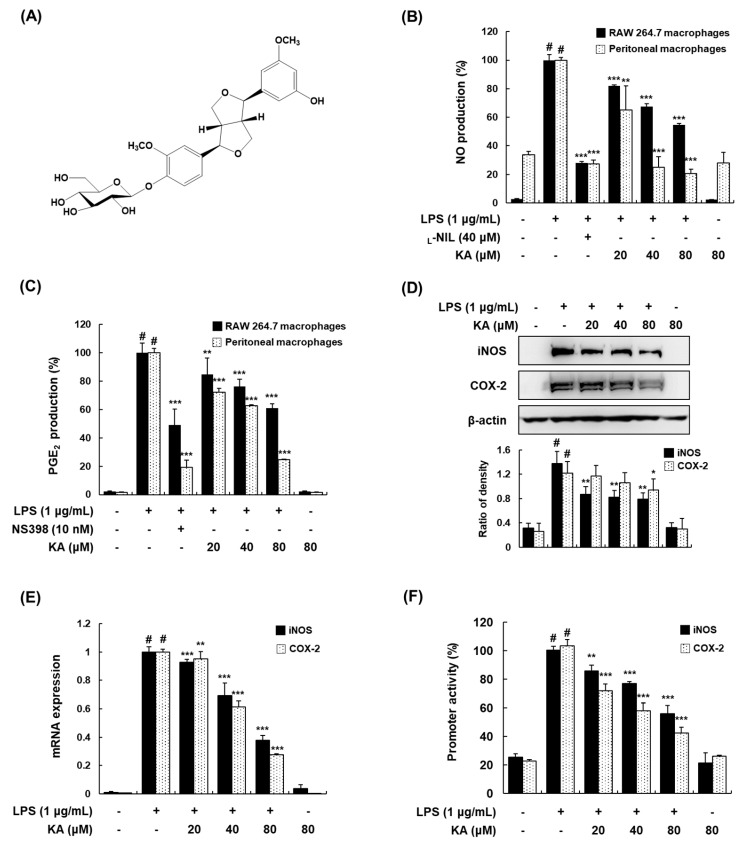
Effects of koreanaside A (KA) on nitric oxide (NO) and prostaglandin E_2_ (PGE_2_) production and the expression of inducible nitric oxide synthase (iNOS) and cyclooxygenase-2 (COX-2) and their promoter activity in lipopolysaccharide (LPS)-induced RAW 264.7 macrophages. (**A**) Structure of KA. (**B**,**C**) Cells were pretreated with KA (20, 40, or 80 μM) for 1 h and then stimulated with LPS (1 μg/mL) for 24 h. NO and PGE_2_ production in the culture media were quantified using the Griess assay and an enzyme immunoassay (EIA) kit, respectively. Positive controls for NO and PGE_2_ production were _L_-NIL (40 μM) and NS-398 (10 nM), respectively. (**D**) Lysates were prepared from cells pretreated with KA (20, 40, or 80 μM) for 1 h and then stimulated with LPS for 24 h. Total cellular proteins were prepared and resolved by SDS-PAGE, and detected using specific iNOS and COX-2 antibodies. β-actin was used as an internal control. (**E**) Total RNA was prepared from cells stimulated with LPS for 4 h in the presence of KA (20, 40, or 80 μM) for qRT-PCR analysis of iNOS and COX-2. The levels of iNOS and COX-2 were adjusted by β-actin expression. (**F**) Cells were transiently transfected with a pGL3-iNOS or a pGL3-COX-2 promoter vector, and a phRL-TK vector was used as an internal control. Cells were pretreated with KA (20, 40, or 80 μM) for 1 h and then stimulated with LPS for 18 h. The values shown are the mean ± SDs of three independent experiments. ^#^ *P* < 0.05 vs. the control group; ^*^ *P* < 0.05, ^**^ *P* < 0.01, ^***^ *P* < 0.001 vs. LPS-stimulated cells.

To determine the anti-inflammatory effects of KA, we examined the effects of KA (20, 40, or 80 μM) on the LPS-induced production of nitroc oxide (NO) and prostaglandin E_2_ (PGE_2_) in RAW 264.7 and peritoneal macrophages. In these cells, KA was found to significantly inhibit the LPS-induced production of NO and PGE_2_ in a concentration-dependent manner (Figure 1B,C). _L_-NIL and NS-398 were used as positive inhibitors of NO and PGE_2_ production, respectively. The inhibitory effects of KA on NO and PGE_2_ production were not caused by its nonspecific cytotoxicity, because KA had no effect on cell viability, as determined by MTT assay up to 80 μM (data not shown). To determine whether the inhibitory effects of KA on NO and PGE_2_ production are related to inducible nitric oxide synthase (iNOS) and cyclooxygenase-2 (COX-2) expression, we evaluated iNOS and COX-2 protein and mRNA expression by Western blotting and qRT-PCR, respectively. The expression of iNOS and COX-2 was markedly upregulated by LPS, and KA (20, 40, or 80 μM) inhibited this upregulation (Figure 1D,E). In addition, KA (20, 40, or 80 μM) considerably reduced the transcriptional activities of the iNOS and COX-2 gene promoters in a concentration-dependent manner (Figure 1F).

### 3.2. KA Inhibits LPS-Induced IL-6 and TNF-α Production and Expression in LPS-Induced RAW 264.7 Macrophages

**Figure 2:**
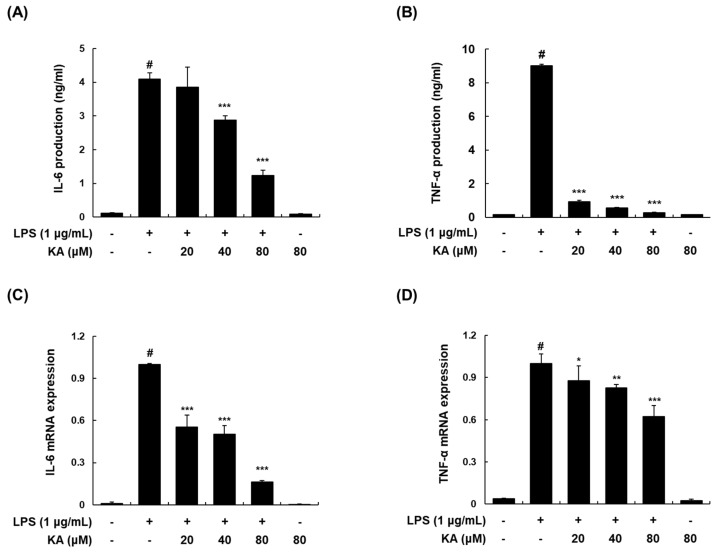
Effects of KA on the LPS-induced production and mRNA expression of interleukin (IL)-6 and tumor necrosis factor-α (TNF-α) in RAW 264.7 macrophages. (**A**,**B**) Cells were pretreated with KA (20, 40, or 80 μM) for 1 h prior to stimulation with LPS (1 μg/mL) for 6 h. IL-6 and TNF-α in the culture media were quantified using EIA kits, respectively. (**C**,**D**) Total RNA was prepared from cells pretreated with KA (20, 40, or 80 μM) for 1 h and then stimulated with LPS for 2 h. The mRNA levels of IL-6 and TNF-α were determined by qRT-PCR. The levels of IL-6 and TNF-α were adjusted by β-actin expression. The values shown are the mean ± SD of three independent experiments. ^#^ *P* < 0.05 vs. the control group; ^*^ *P* < 0.05, ^**^ *P* < 0.01, ^***^ *P* < 0.001 vs. LPS-stimulated cells.

We examined the effects of KA on the production and mRNA expression of IL-6 and TNF-α in LPS-induced macrophages. Pretreatment with KA (20, 40, or 80 μM) reduced the LPS-stimulated production and mRNA expression of IL-6 and TNF-α in a concentration-dependent manner (Figure 2A–D).

### 3.3. KA Inhibits the AP-1 Signaling Pathway in LPS-Induced RAW 264.7 Macrophages

**Figure 3:**
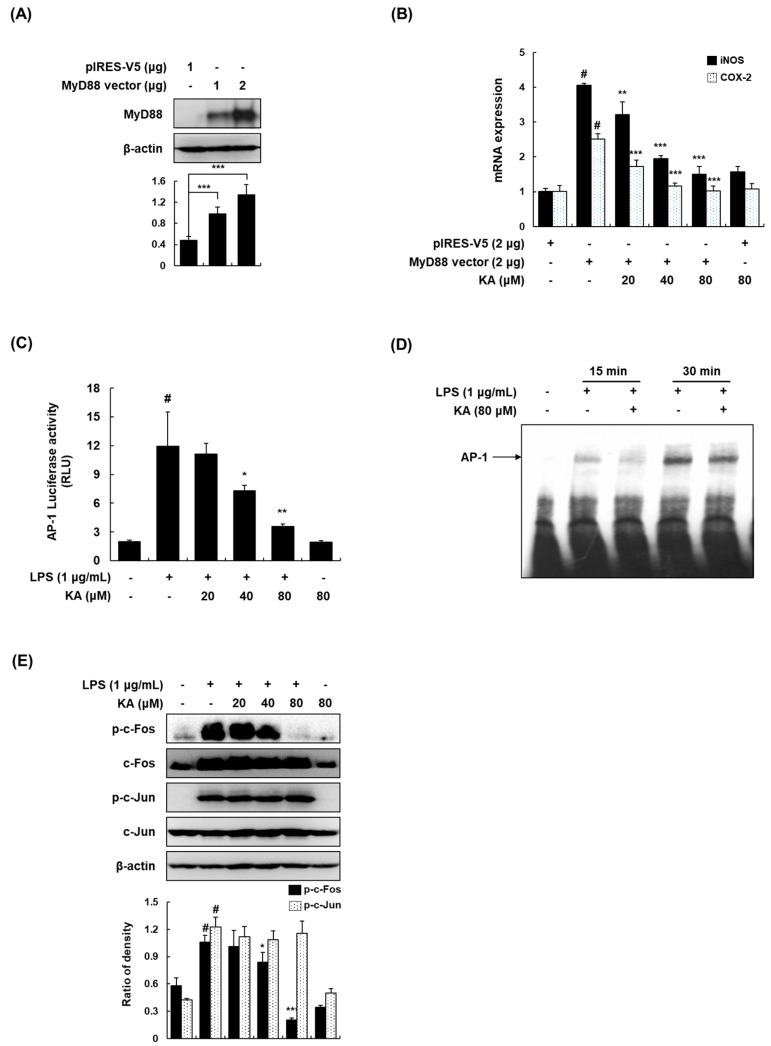
Effects of KA on the activator protein-1 (AP-1) signaling pathway in LPS-stimulated RAW 264.7 macrophages. (**A**) Cells were transfected with V5-MyD88 expression vector for 48 h. Total cellular proteins were prepared, resolved by SDS-PAGE, and detected using specific MyD88 antibodies. β-actin was used as an internal control. (**B**) Cells were transfected with V5-MyD88 expression vector for 48 h and then treated with KA (20, 40, or 80 μM) for 2 h. The mRNA levels of iNOS and COX-2 were determined by qRT-PCR. The levels of iNOS and COX-2 were adjusted by β-actin expression. ^#^ *P* < 0.05 vs. the V5-control group; ^**^ *P* < 0.01, ^***^ *P* < 0.001 vs. MyD88 overexpression cells. (**C**) Cells were transiently transfected with pAP-1-luc reporter; phRL-TK vector was used as an internal control. Cells were pretreated with KA (20, 40, or 80 μM) for 1 h and then stimulated with LPS (1 μg/mL) for 18 h. Luciferase activity levels were determined using the Promega Luciferase Assay System. (**D**) Nuclear extracts were prepared from cells stimulated with LPS at 15 and 30 min in the presence of KA (80 μM), and the DNA-binding activity of AP-1 was analyzed by electrophoretic mobility shift assay (EMSA). (**E**) Cells were pretreated with KA (20, 40, or 80 μM) for 1 h and then stimulated with LPS for 30 min. Total cellular proteins were prepared, resolved by SDS-PAGE, and detected using specific p-c-Fos, c-Fos, p-c-Jun, and c-Jun antibodies. β-actin was used as an internal control. Each experiment was performed three times, and similar results were obtained in each experiment. ^#^ *P* < 0.05 vs. the control group; ^*^ *P* < 0.05, ^**^ *P* < 0.01, ^***^ *P* < 0.001 vs. LPS-stimulated cells.

As pro-inflammatory markers such as iNOS and COX-2 were reduced by KA in LPS-induced RAW 264.7 macrophages, we examined the underlying molecular mechanisms involved in LPS-induced RAW 264.7 macrophages. MyD88 triggers the LPS-induced inflammatory responses through activating transcription factor AP-1 and NF-κB [10]. We transfected RAW 264.7 cells with the TLR4 adaptor molecule MyD88 to induce inflammatory signaling (Figure 3A), and determined the inhibitory effects of KA on MyD88-induced inflammatory responses. The MyD88-induced mRNA expression of iNOS and COX-2 was markedly reduced by KA (Figure 3B), indicating that KA suppressed the MyD88-dependent TLR4 pathway. To identify the exact target transcription factor of KA, an AP-1 reporter gene assay was performed. As shown in Figure 3C, KA (20, 40, or 80 μM) inhibited AP-1-dependent luciferase activity in a concentration-dependent manner. The DNA-binding activity of AP-1 was significantly increased by LPS, and KA (80 μM) attenuated the LPS-induced DNA binding of AP-1 at 15 and 30 min (Figure 3D). In addition, we investigated whether KA blocks the phosphorylation of AP-1 components (c-Fos and c-Jun). As shown in Figure 3E, KA treatment abrogated the LPS-induced phosphorylation of c-Fos; however, KA had no effect on c-Jun phosphorylation.

### 3.4. KA Inhibits the NF-κB Signaling Pathway in LPS-Induced RAW 264.7 Macrophages

**Figure 4:**
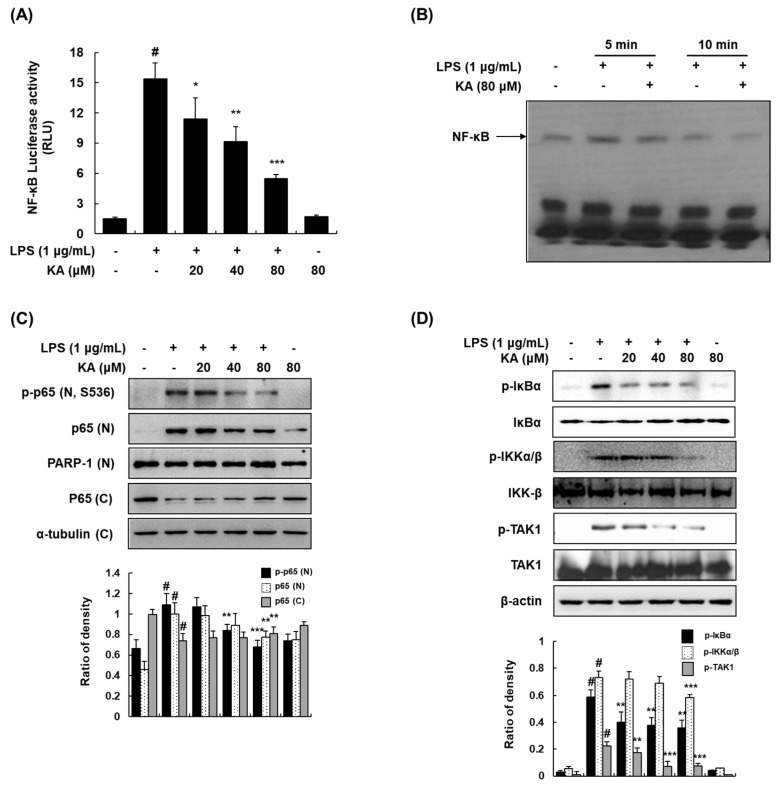
Effects of KA on the nuclear factor-kappa B (NF-κB) signaling pathway in LPS-induced RAW 264.7 macrophages. (**A**) Cells were transiently transfected with pNF-κB-luc reporter; phRL-TK vector was used as an internal control. Cells were pretreated with KA (20, 40, or 80 μM) for 1 h and then stimulated with LPS (1 μg/mL) for 18 h. Luciferase activity levels were determined using the Promega Luciferase Assay System. (**B**) Nuclear extracts were prepared from cells stimulated with LPS at 5 and 10 min in the presence of KA (80 μM), and the DNA-binding activity of NF-κB was analyzed by EMSA. (**C**) Nuclear and cytosolic proteins were extracted from cells pretreated with KA (20, 40, or 80 μM) for 1 h and then stimulated with LPS for 5 min. The nuclear and cytosolic proteins were prepared, resolved by SDS-PAGE, and detected using specific p-p65 and p65 antibodies. Poly(ADP-ribose) polymerase-1 (PARP-1) and α-tubulin were used as internal controls for the nuclear and cytosolic fractions, respectively. (**D**) Cells were pretreated with KA (20, 40, or 80 μM) for 1 h and then stimulated with LPS for 5 min. Total cellular proteins were prepared, resolved by SDS-PAGE, and detected using specific p-IκBα, inhibitor of κB (IκBα), p-IKKα/β, IκB kinase (IKK-β), p-TAK1, and TGF-β-activated kinase 1 (TAK1) antibodies. β-actin was used as an internal control. Each experiment was performed three times, and similar results were obtained in each experiment. ^#^ *P* < 0.05 vs. the control group; ^*^ *P* < 0.05, ^**^ *P* < 0.01, ^***^ *P* < 0.001 vs. LPS-stimulated cells.

Since KA was involved in the MyD88-dependent pathway, we determined whether KA downregulates the NF-κB activation by estimating the LPS-induced NF-κB-dependent transcriptional activity and DNA-binding activity. KA (20, 40, or 80 μM) inhibited NF-κB-dependent luciferase activity in a concentration-dependent manner (Figure 4A). LPS stimulation resulted in a significant increase in the DNA-binding activity of NF-κB as determined by EMSA, and KA reduced the LPS-induced DNA binding of NF-κB at 5 and 10 min (Figure 4B). As shown in Figure 4C, KA also inhibited the LPS-induced phosphorylation and nuclear translocation of p65. In addition, we examined whether KA inhibits the phosphorylation and degradation of IκBα in LPS-induced RAW 264.7 cells. Pretreatment with KA (20, 40, or 80 μM) significantly inhibited the phosphorylation and degradation of IκBα. The phosphorylation of IKKα/β, upstream kinases of IκB, was markedly inhibited by KA. As TAK1 regulates the phosphorylation of IKKα/β by LPS in the NF-κB pathway [25], we further investigated the effect of KA on the phosphorylation of TAK1 in LPS-induced RAW 264.7 cells. The LPS-induced phosphorylation of TAK1 was suppressed by KA (Figure 4D).

### 3.5. KA Inhibits JAK/STAT Phosphorylation in LPS-Induced RAW 264.7 Macrophages

**Figure 5:**
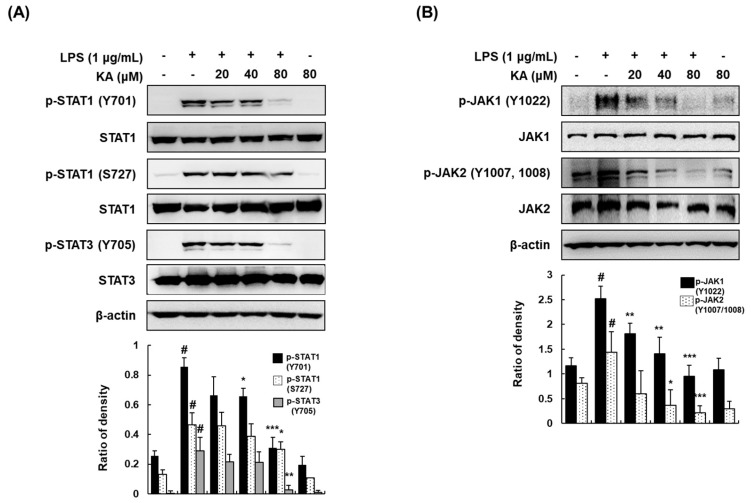
Effects of KA on Janus kinase/signal transducers and activators of transcription (JAK/STAT) phosphorylation in LPS-induced RAW 264.7 macrophages. (**A**,**B**) Cells were pretreated with KA (20, 40, or 80 μM) for 1 h, and then stimulated with LPS (1 μg/mL) for 120 min (STATs) or 60 min (JAKs). Total cellular proteins were prepared, resolved by SDS-PAGE, and detected using specific p-STAT1 (Y701 and S727), p-STAT3 (Y705), STAT1, STAT3, p-JAK1 (Y1022), JAK1, p-JAK2 (Y1007/1008), and JAK2 antibodies. β-actin was used as an internal control. Each experiment was performed three times, and similar results were obtained in each experiment. ^#^ *P* < 0.05 vs. the control group; ^*^ *P* < 0.05, ^**^ *P* < 0.01, ^***^ *P* < 0.001 vs. LPS-stimulated cells.

As the Janus kinase/signal transducers and activators of transcription (JAK/STAT) signaling pathway is involved in the pathogenesis of several diseases, including IBD [26], we evaluated the effects of KA on the JAK/STAT signaling pathway. KA (20, 40, or 80 μM) suppressed the phosphorylation of STAT1 (Y701 and S727) and STAT3 (Y705) (Figure 5A). A previous study reported that phosphorylation at the tyrosine residue of STAT proteins results from the activation of JAKs [27]. Thus, we examined the phosphorylation of JAK1 and JAK2 in LPS-induced macrophages. Likewise, KA (20, 40, or 80 μM) effectively reduced the phosphorylation of JAK1 (Y1022) and JAK2 (Y1007/1008) (Figure 5B).

### 3.6. KA Attenuates Colitis Severity, Colon Shortening, and Spleen Enlargement in a DSS-Induced Acute Colitis Model

**Figure 6:**
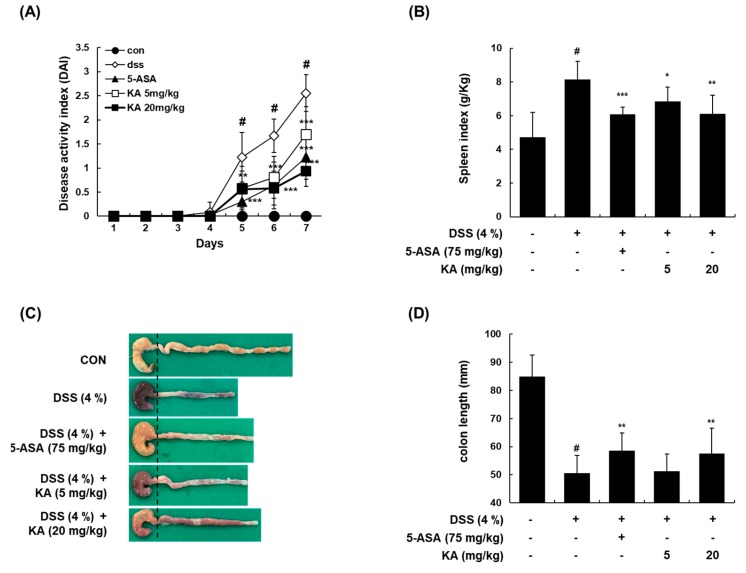
Ameliorative effects of KA on dextran sulfate sodium (DSS)-induced colitis in mice. (**A**) Mice were administered with 4% DSS in drinking water for 7 days with KA (5 or 20 mg/kg/day, i.p.). 5-ASA (75 mg/kg/day, p.o.) was used a positive control. DAI were evaluated daily. (**B**–**D**) On day 7, the mice were sacrificed, and their colon and spleen were obtained. The colon length and spleen weight of each mouse were evaluated. Values are the mean ± SD (*n* = 12); ^#^ *P* < 0.05 vs. the vehicle-treated control group; ^*^ *P* < 0.05, ^**^ *P* < 0.01, ^***^ *P* < 0.001 vs. the DSS-treated group.

To investigate the anti-inflammatory effects of KA in vivo, a mouse model of colitis induced by 4% DSS in drinking water for 7 days was used. For the positive control, 5-ASA (75 mg/kg, p.o.) was used. Following the induction of colitis, the combinational DAI, including body weight loss, stool consistency, and the appearance of occult fecal blood, was significantly increased after 5 days in the DSS-treated group compared with the vehicle-treated control group. However, the administration of KA (5 or 20 mg/kg) significantly decreased DAI values. The administration of 5-ASA showed similar suppressive effects (Figure 6A). After 7 days of DSS treatment, the colon length of the DSS-treated group was significantly shorter than that of the vehicle-treated control group (50.54 ± 6.35 mm versus 84.97 ± 7.61 mm, *P* < 0.001). However, the administration of KA or 5-ASA reduced the shortening of the colon length (DSS-treated group versus DSS + 5-ASA-treated group, 50.54 ± 6.35 mm versus 58.51 ± 6.28 mm, *P* < 0.01; DSS-treated group versus DSS + KA (5 mg/kg)-treated group, 50.54 ± 6.35 mm versus 51.22 ± 6.10 mm, *P* < 0.05; DSS-treated group versus DSS + KA (20 mg/kg)-treated group, 50.54 ± 6.35 mm versus 57.49 ± 9.10 mm, *P* < 0.01) (Figure 6C,D). The spleen is important for protecting the body from invading pathogens and plays a vital role in the adaptive response to inflammation [28]. In this study, the spleen index (spleen weight (g)/ body weight (kg)) of the DSS-treated group was higher than that of the vehicle-treated control group (8.13 ± 1.09 g/kg versus 4.71 ± 1.49 g/kg, *P* < 0.001). Treatment with 5-ASA (75 mg/kg) or KA (5 or 20 mg/kg) significantly inhibited spleen hypertrophy in DSS-induced colitis mice (DSS-treated group versus DSS + 5-ASA-treated group, 8.13 ± 1.09 g/kg versus 6.07 ± 0.45 g/kg, *P* < 0.001; DSS-treated group versus DSS + KA (5 mg/kg)-treated group, 8.13 ± 1.09 g/kg versus 6.85 ± 0.86 g/kg, *P* < 0.05; DSS-treated group versus DSS + KA (20 mg/kg)-treated group, 8.13 ± 1.09 g/kg versus 6.10 ± 1.12 g/kg, *P* < 0.01) (Figure 6B).

### 3.7. KA Decreases Colon Tissue Injury and Restores Epithelial Barrier mRNA Expression in Mice with DSS-Induced Colitis

**Figure 7:**
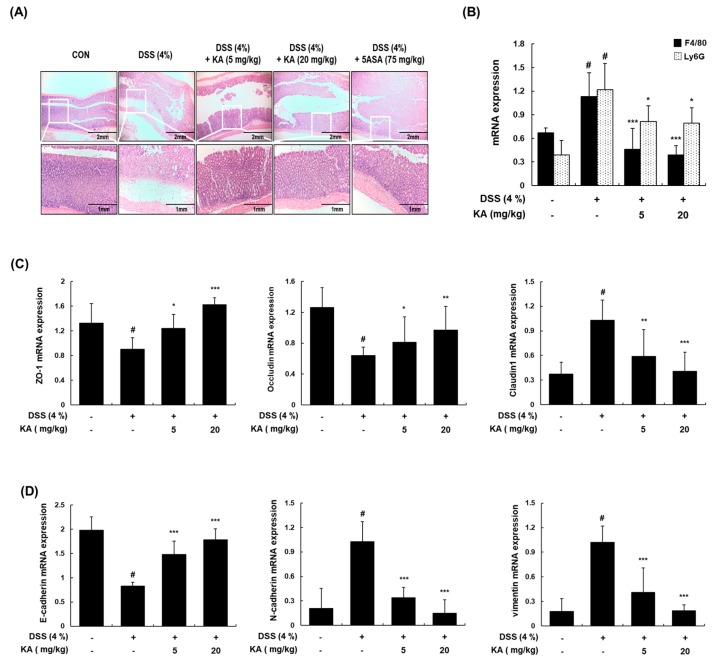
Effects of KA on histological changes and the expression of tight junction (TJ)- and epithelial–mesenchymal transition (EMT)-related markers in DSS-induced colonic tissues. (**A**) Representative sections of colon tissues from mice administrated with 4% DSS in drinking water for 7 days with KA (5 or 20 mg/kg/day, i.p.). 5-ASA (75 mg/kg/day, p.o.) was used as a positive control. Histological changes were determined by H&E staining. (**B**–**D**) Total RNAs were prepared from DSS-induced colon tissues and analyzed for the mRNA expression of F4/80, Ly6G, zona occludens (ZO-1), occludin, claudin1, E-cadherin, N-cadherin, and vimentin by qRT-PCR. The levels of F4/80, Ly6G, ZO-1, occludin, claudin1, E-cadherin, N-cadherin, and vimentin were adjusted by β-actin expression. Values are the mean ± SDs (*n* = 12); ^#^ *P* < 0.05 vs. the vehicle-treated control group; ^*^ *P* < 0.05, ^**^ *P* < 0.01, ^***^ *P* < 0.001 vs. the DSS-treated group.

To determine the inhibitory effects of KA on the severity of colonic inflammation and ulceration, histopathological evaluation was performed by examining colon sections after hematoxylin and eosin (H&E) staining using a light microscope. The colon tissues of the DSS-treated group showed the broad destruction of the mucosal layer, ulceration, and crypt loss. However, KA (5 or 20 mg/kg) administration protected crypt structures and reduced the pathological signs of colon tissue damage (Figure 7A). As the infiltration of monocytes/macrophages and neutrophils is important in the pathogenesis of DSS-induced colitis [29], we examined the mRNA expression of F4/80 and Ly6G (well-known macrophage and neutrophil biomarkers, respectively). The inflammatory cell infiltration markers were markedly increased in the DSS-treated group compared with the vehicle-treated control group. However, treatment with KA (5 or 20 mg/kg) suppressed the DSS-induced mRNA expression of F4/80 and Ly6G (Figure 7B). The intestinal TJ may play a key role in epithelial barrier regulation and inflammatory cell migration [3,30]. In this study, we evaluated the mRNA expression of ZO-1, occludin, and claudin-1 in the colon tissues by qRT-PCR. The downregulation of ZO-1 and occludin and upregulation of claudin-1 mRNA levels were detected in the DSS-treated group compared with the vehicle-treated control group. The DSS + KA (5 or 20 mg/kg)-treated group showed the restored mRNA levels of ZO-1, occludin, and claudin-1 (Figure 7C). It is known that the EMT marker E-cadherin is downregulated, whereas N-cadherin and vimentin are upregulated during the loss of cell adhesion [31]. To determine the effects of KA on these EMT markers, we investigated the mRNA expression of E-cadherin, N-cadherin, and vimentin in colon tissues by qRT-PCR. The mRNA levels of E-cadherin were decreased, whereas those of N-cadherin and vimentin were markedly increased in the DSS-treated group compared with the vehicle-treated control group. However, treatment with KA (5 or 20 mg/kg) significantly restored the mRNA expression of E-cadherin, N-cadherin, and vimentin (Figure 7D).

### 3.8. KA Inhibits iNOS, COX-2, and Inflammatory Cytokine mRNA Expression through AP-1, NF-κB, and STAT1/3 Inactivation in DSS-Induced Colon Tissues

**Figure 8:**
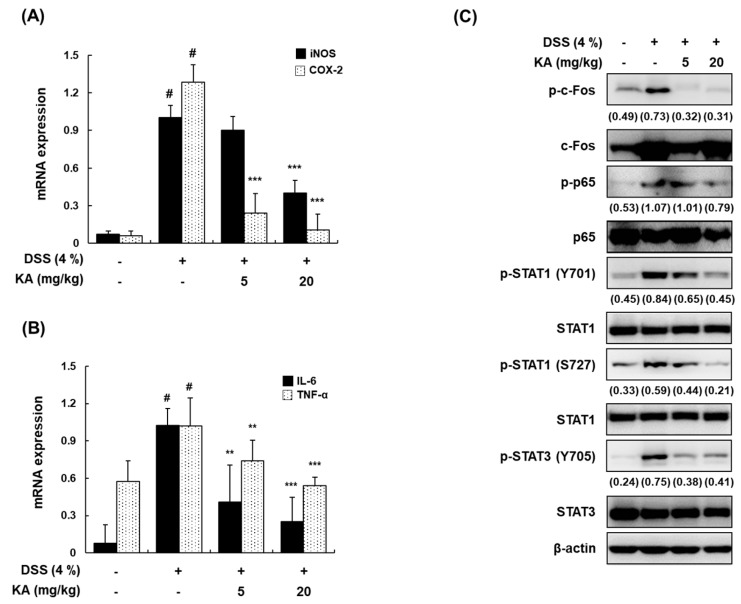
Inhibitory effects of KA on pro-inflammatory mediators and AP-1, NF-κB, and STAT1/3 activation in DSS-induced colon tissues. (**A**,**B**) Total RNAs were prepared from DSS-induced colon tissues and analyzed for the mRNA expression of iNOS, COX-2, IL-6, and TNF-α by qRT-PCR. The levels of iNOS, COX-2, IL-6, and TNF-α were adjusted by β-actin expression. Values are the mean ± SDs (*n* = 12); ^#^ *P* < 0.05 vs. the vehicle-treated control group; ^*^ *P* < 0.05, ^**^ *P* < 0.01, ^***^ *P* < 0.001 vs. the DSS-treated group. (**C**) Colon tissues were homogenized after 7 days of DSS treatment, and whole proteins were prepared for Western blotting to detect the protein expression of p-c-Fos, c-Fos, p-p65, p65, p-STAT1 (Y701), STAT1, p-STAT (S727), p-STAT3 (Y705), and STAT3 antibodies. β-actin was used as an internal control. Values in brackets means the relative density of a representative image.

In DSS-induced ulcerative colitis, it is well known that the expression of iNOS and COX-2 is upregulated, followed by the increased expression of pro-inflammatory cytokines such as IL-6 and TNF-α [32,33]. The mRNA expression of iNOS, COX-2, IL-6, and TNF-α in colon tissues was upregulated by DSS. However, treatment with KA (5 or 20 mg/kg) significantly inhibited these upregulations (Figure 8A,B). Furthermore, we examined the activation of c-Fos, p65, STAT1 (Y701 and S727), and STAT3 (Y705) in colon tissues by Western blotting. The phosphorylation of c-Fos, p65, STAT1 (Y701 and S727), and STAT3 (Y705) was greatly increased by DSS; however, treatment with KA (5 or 20 mg/kg) markedly attenuated DSS-induced phosphorylation (Figure 8C).

## 4. Discussion

In inflammation, macrophages mediate innate immune responses, which are the first line of defense against several pathogens and various chronic inflammatory diseases, by producing inflammatory mediators including NO, PGE_2_, TNF-α, and IL-6 [9,34]. Phytochemicals are a major group of drug candidates that may be used for the suppression of the inflammatory response, and extensive efforts have been made to identify effective anti-inflammatory phytochemicals. As part of our ongoing efforts, we isolated seven lignan compounds (koreanaside A, koreanaside B, koreanaside C, matairesinol, matairesinoside, arctiin, and arctigenin) from the flowers of *F. koreana* and evaluated their anti-inflammatory effects in LPS-induced macrophages. Although the radical scavenging activity and VCAM-1 inhibitory effect of KA have been reported [22], the anti-inflammatory effects of the seven compounds have not been reported. The inhibitory effect of KA on LPS-induced TNF-α production (IC_50_; 16.3 ± 1.0 μM) was stronger than that of other lignan compounds (data not shown). Furthermore, KA inhibited the activation of AP-1, NF-κB, and JAK/STAT and the subsequent induction of pro-inflammatory mediators in LPS-induced macrophages and DSS-induced colitis mice. The anti-inflammatory effects of KA were consistent with the effects on pro-inflammatory mediators, i.e., NO, PGE_2_, IL-6, and TNF-α, in LPS-induced macrophages; they are known to be involved in the regulation of immune reactions, inflammation, and, in some cases, colitis and death [35]. We found that KA inhibited the LPS-induced production of NO and PGE_2_ by suppressing the transcription of iNOS and COX-2, which resulted in the downregulation of protein and mRNA expression. In addition, KA inhibited the LPS-induced production and mRNA expression of IL-6 and TNF-α.

TLR4, a member of the TLR family, is activated by LPS and recruits adaptor proteins such as MyD88 and TIR-domain-containing adapter-inducing interferon-β (TRIF)-related adaptor molecule (TRAM), initiating the MyD88-dependent pathway. The activation of Myd88 recruits two IL-1R-associated kinases (IRAK1 and IRAK4), leading to TNF receptor-associated factor 6 (TRAF6) activation. The activated TRAF6 is ubiquitinated and recruited to the TAK1/TAB protein complex, activating the signaling cascades of AP-1, NF-κB, and MAPKs [36,37,38]. To better elucidate the anti-inflammatory responses of KA at the molecular level, inflammatory responses were induced by MyD88 overexpression. The mRNA expression of iNOS and COX-2 induced by MyD88 overexpression was significantly inhibited by KA. The results indicated that KA could exert anti-inflammatory activities by downregulating the MyD88-dependent pathway. Therefore, we determined the inhibitory effects of KA on MyD88-dependent transcription factors such as AP-1 and NF-κB in LPS-induced RAW 264.7 macrophages. In a normal state, macrophages express low levels of AP-1 subunits (c-Fos and c-Jun); however, their expression can be increased when stimulated by LPS [39]. Therefore, we examined the effects of KA on AP-1 transcriptional and DNA-binding activities to clarify the molecular mechanisms that modulate pro-inflammatory mediators. Our results showed that KA inhibited the LPS-induced transcriptional activity and DNA-binding activity of AP-1. In addition, KA had an inhibitory effect on the LPS-induced nuclear accumulation of c-Fos but not c-Jun, indicating the inhibition of the AP-1 pathway.

NF-κB is known to regulate genes involved in cell survival and coordinate the expression of pro-inflammatory mediators [15]. In this study, we found that KA inhibited the LPS-induced transcriptional and DNA-binding activities of NF-κB, as shown by reporter gene assay and EMSA, respectively. To identify the mechanisms involved in the inhibition of NF-κB activity by KA, we examined the effect of KA on NF-κB signaling. Inactive NF-κB binds to IκBα, which is an inhibitory protein in the cytoplasm; however, after cellular stimulation, IκBα is phosphorylated at certain serine residues through polyubiquitination and proteasomal degradation, freeing NF-κB and allowing translocation to the nucleus [14]. KA was found to inhibit the LPS-induced phosphorylation and degradation of IκBα and p65 in nuclear fractions. It is known that the phosphorylation of IκB is regulated by IKKα/β, which is further regulated by upstream factors such as TAK1 [25]. In the present study, KA inhibited the phosphorylation of IKKα/β and TAK1, suggesting that KA could suppress NF-κB activation via the downregulation of the TAK1-mediated NF-κB pathway in LPS-induced RAW 264.7 macrophages. TAK1 can phosphorylate MAPKs and the IKK complex, and the MAPK cascade leads to the production of pro-inflammatory cytokines by regulating the activation of inflammatory transcription factors [40,41]. To further determine whether KA downregulates pro-inflammatory mediators by inhibiting MAPK pathways, we investigated the effect of KA on the phosphorylation of JNK, ERK, and p38 in LPS-induced RAW 264.7 macrophages. Our results indicated that KA suppressed LPS-induced ERK phosphorylation, but not JNK and p38 phosphorylation (Supplementary Figure S1).

Signaling through the JAK/STAT pathway is critical for the inflammatory cascades in IBD pathogenesis [26]. LPS and IFN-γ induce the phosphorylation of the JAK family members, particularly JAK1 and JAK2, at tyrosine residues, which are receptor kinases involved in STAT1/3 phosphorylation [42,43]. In the present study, we demonstrated that KA inhibited the phosphorylation of STAT1 (Y701 and S727) and STAT3 (Y705) and their receptor tyrosine kinases JAK1 (Y1022) and JAK2 (Y1007/1008). Collectively, our results suggest that the anti-inflammatory effects of KA may be attributed to the suppression of STAT1 and STAT3 through the inhibition of JAK1 and JAK2. Furthermore, we demonstrated that the suppressive effect of KA on LPS-induced STAT1 phosphorylation was stronger at Y701 than at S727. It is possible that KA did not affect the activation of p38, because STAT1 phosphorylation at S727 is enhanced by p38 in LPS stimulation [44]. Based on the results, the inhibition on the expression of pro-inflammatory mediators by KA might be mediated by the inactivation of NF-κB, AP-1, and JAK/STAT in LPS-activated macrophages.

Murine models of intestinal inflammation have been widely used to investigate the regulatory mechanisms involved in reducing inflammation and restoring intestinal homeostasis. In this study, KA treatment considerably attenuated body weight loss, diarrhea, bloody feces, colon shortening, and histological damage in a DSS-induced colitis model. Inflammatory cells such as neutrophils and macrophages have been found to be increased in the colon and secrete various inflammatory mediators, playing critical roles in DSS-induced colitis disease [7]. Our findings showed that the infiltration of activated macrophages and neutrophils was markedly increased, and that pretreatment with KA reduced these effects. In agreement with these results, KA reduced the mRNA levels of iNOS, COX-2, TNF-α, and IL-6 in colon tissues. The pathological features of IBD are closely associated with exaggerated inflammation and the subsequent destruction of the intestinal epithelium [45]. The intestinal barrier regulates macromolecule trafficking between the lumen and internal milieu, and protects the host by preventing harmful solutes, microorganisms, toxins, and luminal antigens from impairing the body defense mechanism [46]. This barrier is formed by the interaction of various barrier components such as intercellular TJ proteins [47]. Our results showed that the expression of three major TJ proteins (ZO-1, occludin, and claudin-1) was regulated by KA. Therefore, the effects of KA on the epithelial barrier could be mediated by maintaining the expression of TJ proteins, reducing the severity of gut inflammation. During the EMT, proteins such as E-cadherin and α-catenin are downregulated, and mesenchymal cell-specific marker proteins including N-cadherin, vimentin, and fibronectin are upregulated [48]. We found that treatment with KA restored the mRNA levels of E-cadherin, N-cadherin, and vimentin in DSS-induced colon tissues. These results suggest that KA suppressed not only colitis-induced inflammatory cell infiltration, but also the functional disruption of the intestinal barrier by modulating the expression of TJ- and EMT-related proteins, thereby decreasing the severity of gut inflammation. Consistent with the results for activated macrophages, Western blot analysis indicated that pretreatment with KA in colon tissues inhibited the phosphorylation of c-Fos, p65, and STAT1/3.

## 5. Conclusions

**Figure 9:**
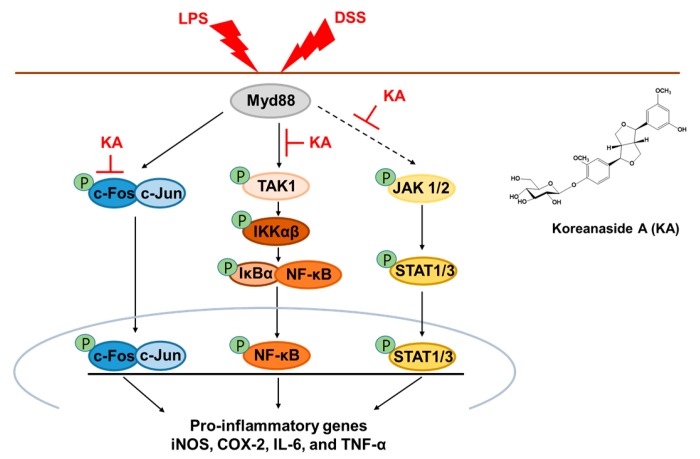
Proposal of molecular mechanism for anti-inflammatory and anti-colitic effects of KA.

In summary, our study revealed that KA inhibited LPS-induced pro-inflammatory mediators through suppression of the AP-1, NF-κB, and STAT1/3 signaling pathway via TAK1 and JAK1/2 inactivation in activated macrophages. Furthermore, the anti-colitic effects of KA may be associated with the maintenance of a TJ and EMT network and inflammatory modulation via the AP-1, NF-κB, and STAT1/3 inactivation signal transduction pathways (Figure 9). Therefore, our findings indicated that KA administration may be a potential treatment option for IBDs such as colitis.

## Supplementary Materials

The following are available online at https://www.mdpi.com/2073-4409/8/10/1163/s1, Figure S1: Effects of KA on MAPKs in LPS-induced RAW 264.7 macrophages. (A) Cells were pretreated with KA (20, 40, or 80 μM) for 1 h, and then stimulated with LPS (1 μg/mL) for 10 min. Total cellular proteins were prepared and resolved by SDS-PAGE, transferred onto PVDF membranes, and detected using specific p-ERK, ERK, p-JNK, JNK, p-p38, and p38 antibodies. β-actin was used as internal control. The experiment was repeated three times, and similar results were obtained. Table S1: List of primers.
