# Supplementary material for: Anti-Inflammatory Mechanisms of Koreanaside A, a Lignan Isolated from the Flower of Forsythia koreana, against LPS-Induced Macrophage Activation and DSS-Induced Colitis Mice: The Crucial Role of AP-1, NF-κB, and JAK/STAT Signaling
Source: Cells. 2019 Sep 27;8(10):1163. doi: 10.3390/cells8101163 (PMC6829247; doi:10.3390/cells8101163)
Supplement: Supplementary file 1 [file cells-08-01163-s001.pdf]

Supplementary Table 1. List of primers.

Gene		Sequence
IL-6	Forward	GAGGATACCACTCCCAACAGACC
	Reverse	AAGTGCATCATCGTTGTTCATACA
TNF-α	Forward	AGCACAGAAAGCATGATCCG
	Reverse	CTGATGAGAGGGAGGCCATT
iNOS	Forward	AATGGCAACATCAGGTCGGCCATCACT
	Reverse	GCTGTGTGTCACAGAAGTCTCGAACTC
COX-2	Forward	GGAGAGACTATCAAGA TAGT
	Reverse	ATGGTCAGTAGACTTTTACA
F4/80	Forward	AGGACTGGAAGCCCATAGCCAA
	Reverse	GCATCTAGCAATGGACAGCTG
Ly6G	Forward	AGACTTCCTGCAACACAACT
	Reverse	TGCAACAGTAAGTCTTCACG
ZO-1	Forward	GCCTGTAAGAGAGGATTCCT
	Reverse	TGTTTCAGGCGAAAGGTAAG
Occludin	Forward	TGGCGGATATACAGACCCAA
	Reverse	CGATCGTGGCAATAAACACC
Claudin1	Forward	CTGTGGATGTCCTGCGTTTC
	Reverse	TCATGCACTTCATGCCAATG
N-cadherin	Forward	GGCCTTAAAGCTGCTGACAA
	Reverse	GGTCCCCAGTCATTCAGGTA
E-cadherin	Forward	CAGGTCTCCTCATGGCTTTGC
	Reverse	CTTCCGAAAAGAAGGCTGTCC
Vimentin	Forward	ATGCTTCTCTGGCACGTCTT
	Reverse	AGCCACGCTTTCATACTGCT
β-actin	Forward	ATCACTATTGGCAACGAGCG
	Reverse	ATCACTATTGGCAACGAGCG

Supplementary Figure S1

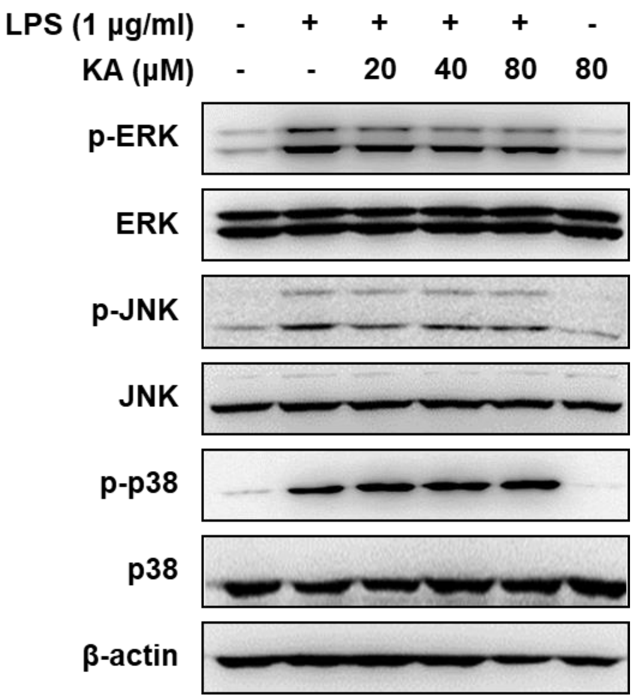

Supplementary Figure 1. Effects of KA on MAPKs activation in LPS-stimulated RAW 264.7 macrophages. (A) Cells were pretreated with KA (20, 40, or 80 μM) for 1 h, and then stimulated with LPS (1 μg/ml) for 10 min. Total cellular proteins were prepared and resolved by SDS-PAGE, transferred onto PVDF membranes, and detected using specific p-ERK, ERK, p-JNK, JNK, p-p38, and p38 antibodies. β-actin was used as internal control. The experiment was repeated three times, and similar results were obtained.
